# Patients Know Best: Qualitative Study on How Families Use Patient-Controlled Personal Health Records

**DOI:** 10.2196/jmir.4652

**Published:** 2016-02-24

**Authors:** Hanna Schneider, Susan Hill, Ann Blandford

**Affiliations:** ^1^ UCLIC UCL London United Kingdom; ^2^ Human-Computer Interaction Group University of Munich (LMU) Munich Germany; ^3^ Great Ormond Street Hospital London United Kingdom; ^4^ UCLIC & Institute of Digital Health UCL London United Kingdom

**Keywords:** electronic health record, patient empowerment, self-determination theory

## Abstract

**Background:**

Self-management technologies, such as patient-controlled electronic health records (PCEHRs), have the potential to help people manage and cope with disease.

**Objective:**

This study set out to investigate patient families’ lived experiences of working with a PCEHR.

**Methods:**

We conducted a semistructured qualitative field study with patient families and clinicians at a children’s hospital in the UK that uses a PCEHR (Patients Know Best). All families were managing the health of a child with a serious chronic condition, who was typically under the care of multiple clinicians. As data gathering and analysis progressed, it became clear that while much of the literature assumes that patients are willing and waiting to take more responsibility for and control over their health management (eg, with PCEHRs), only a minority of participants in our study responded in this way. Their experiences with the PCEHR were diverse and strongly shaped by their coping styles. Theory on coping identifies a continuum of coping styles, from approach to avoidance oriented, and proposes that patients’ information needs depend on their style.

**Results:**

We identified 3 groups of patient families and an outlier, distinguished by their coping style and their PCEHR use. We refer to the outlier as controlling (approach oriented, highly motivated to use PCEHR), and the 3 groups as collaborating (approach oriented, motivated to use PCEHR), cooperating (avoidance oriented, less motivated to use PCEHR), and avoiding (very avoidance oriented, not motivated to use PCEHR).

**Conclusions:**

The PCEHR met the needs of controller and collaborators better than the needs of cooperators and avoiders. We draw on the Self-Determination Theory to propose ways in which a PCEHR design might better meet the needs of avoidance-oriented users. Further, we highlight the need for families to also relinquish control at times, and propose ways in which PCEHR design might support a better distribution of control, based on effective training, ease of use, comprehensibility of data security mechanisms, timely information provision (recognizing people’s different needs), personalization of use, and easy engagement with clinicians through the PCEHR.

## Introduction

### Overview

Patients and parents of patients with complex chronic diseases face social, psychological, and organizational challenges. Many of them need to see 5 or 10 specialty departments of a hospital on a regular basis. They typically need to take medication daily, adhere to a special diet, and perform complex procedures at home such as injections or blood tests. Without prior medical knowledge, it is hard for patients and their families to understand the meaning of diagnoses, test results, and proposed treatments. Hence, they have traditionally had to surrender significant control to their doctors, who are responsible for their care, and to comply with recommendations. Anderson and Funnell [1] refer to this traditional approach to health care as the “acute-care paradigm.”

However, two decades ago a new paradigm set out to change the balance of power: patient empowerment. Its goal is that patients set their own health goals that, then, both clinicians and patients work toward [1,2]. The process of patient empowerment requires patients to learn about their disease, to understand possible treatment options, and to participate in decision making. A popular way to educate patients and to provide them with information and choice is through technology such as Web-based self-management tools [3]. It is hoped that the feeling of control that patients gain through these tools will help them to better cope with and manage their illness [4]. Numerous studies have attempted to measure the effect that health management tools have on patient empowerment. For example, a meta-analysis by Samoocha et al [5] compared 14 randomized control trials that measured the effect of Web-based interventions on patient empowerment and found only small positive effects overall. The 14 studies measured patient empowerment with self-efficacy scales such as the Diabetes Empowerment Scale, assuming that the output of patient empowerment is increased self-efficacy and control. However, other researchers [6,7] have questioned this assumption. Another deficit of many studies on patient empowerment, such as those reviewed by Samoocha et al [5], is that they reveal little about the lived experiences of patients who use a Web-based self-management tool.

This study set out to close this gap: to better understand patients’ lived experience with a patient-controlled electronic health record (PCEHR) and how the use of such a technology may lead to patient empowerment. The study took place in a specialist children’s hospital, so most patients are cared for by a parent, and it was the parent who engaged with the PCEHR. For simplicity, we use the term “patient” to refer to the user of the PCEHR acting on behalf of the patient, and only make a distinction between patient and parent where that distinction is important to the account of their experience.

### Background

As noted in the previous section, the study reported here started with the intention to better understand patient families’ experiences with a PCEHR, based on the assumption that better experience would result in better engagement, and hence greater empowerment. Early data gathering and analysis led us to challenge our own assumptions, to draw on literatures related to coping, self-efficacy, and self-determination, and to shape subsequent data gathering and analysis focusing more directly on the relationship between individual coping styles and experiences of PCEHR use. In this section, we introduce previous work on personal health records (PHRs) and PCEHRs as well as literature related to coping styles.

#### Previous Work on Personal Health Records and PCEHRs

A common definition of a PHR is “an electronic application through which individuals can access, manage and share their health information, and that of others for whom they are authorized, in a private, secure, and confidential environment.” [8]. In this work, we use the term “PCEHR” to refer to a record that gives all rights to the user who can then decide to share (parts of) the record with various health care providers. Researchers have proposed a variety of potential benefits of PHRs and PCEHRs, such as improved patient experience, support for patients with chronic conditions, improved transparency, increased referral rates, and better continuity of care beyond the hospital walls [9]. The main focus of this work is PHRs as a means to foster patient empowerment.

Previous studies of PHR user needs and design recommendations [8-13] have identified unresolved design questions.

##### Should Patients Be Given Immediate Access to Test Results?

While many patients seem to be interested in viewing their PHR [10], it is unclear whether new test results should be displayed immediately or after consultations [9]. While Byczkowski et al [14] found that participants were well prepared and appreciated immediate access, others [11,15] concluded that abnormal results should be discussed with the health care professional first.

##### Should Patients Be Able to Edit the Health Record?

It is unclear whether patients want to edit or add data in their health record, thus exercising control over their PHR [9]. Munir and Boaden [10] found that even though a majority of patients wanted to view their record, most of them did not want to control it. They concluded that patients’ desire to be empowered (in terms of exercising control) varies and depends mainly on age and technical literacy. By contrast, Winkelman et al [11] found that patients welcomed the opportunity to edit and add data.

##### What Are Patients’ Information Needs?

Providing the right amount of information and presenting it in a comprehensible fashion to patients seems to be challenging: Gysels et al [16] found that only some patients felt better informed thanks to a PHR, and Earnest et al [15] and Byczkowski et al [14] found that patients need more explanatory information about relevant disease markers and better presentation of the information. In contrast, Pai et al [17] reported that patient’s felt better informed thanks to the PHR. Recognizing that patients’ information needs depend not only on their condition but also on the context of use, Attfield et al [18] investigated how patients’ information needs vary over time, specifically before and after consultations. Although it is now clear that both the condition and the context of use influence patients’ information needs, other factors still need to be investigated, such as patients’ personal priorities and motivations.

#### Users of PCEHRs: People With Different Coping Styles

Users of a PCEHR include people with a chronic disease, their caregivers, and health care professionals. An important source of variability among our participants that influenced their relationship to the PCEHR was found to be their coping styles.

Although many researchers have attempted to conceptualize coping strategies and styles, the effects of coping on psychological, physiological, and behavioral outcomes are poorly understood [19]. However, much of the coping literature distinguishes between approach- and avoidance-oriented coping [19-23]. *Approach-oriented coping* typically involves information seeking, problem solving, seeking social support, actively attempting to identify benefits in one’s experience, or creating outlets for emotional expression. *Avoidance-oriented coping* typically involves cognitive strategies such as denial and suppression and behavioral strategies such as disengagement.

Many researchers [20] have argued that approach-oriented coping is more effective than avoidance-oriented coping. However, pushing people to take more responsibility and control can be counterproductive. Giving patients control, responsibility, or information when they do not want it can, for example, increase distress [7]. There is an alternative viewpoint that coping strategies are not inherently good or bad; rather, coping styles can be more or less appropriate and effective in certain contexts, depending on, for example, the controllability of a situation [24,25]. Folkman and Moskowitz [19] argue that the focus should be on coping-environment fit and on assessing people’s coping flexibility, defined as their ability to modify their coping according to the situational demands.

Our study prompted us to question whether and how a PCEHR can meet the needs of patients with different coping styles.

##### Facilitating Approach-Oriented Coping With Self-Determination Theory

As noted earlier, people’s coping styles are associated with their motivations. Self-Determination Theory (SDT) [26] offers an account of the circumstances under which people develop intrinsic motivation, that is, their natural tendency to seek out novelty and challenges, to learn, and to extend and exercise their capabilities. According to SDT, motivation can range from amotivation (ie, total lack of motivation) through various degrees of extrinsic motivation to intrinsic motivation. While intrinsic motivation is completely internalized, extrinsic motivation can be more or less shaped by internal factors, and internal motivators are typically stronger than external ones. According to this theory, intrinsic motivation will flourish if basic needs for competence, autonomy, and relatedness are fulfilled [26].

C*ompetence* refers to a feeling of confidence and effectiveness in the domain of behavior in focus. Feelings of competence can, for example, be enhanced when people around the actor provide meaningful positive feedback [27].

*Autonomy* refers to an internal perceived locus of control or regulation by the self. An autonomous individual experiences his or her behavior as self-organized [26].

*Relatedness* refers to a sense of connection with others and belonging. This implies a feeling of being cared for and included within the domain of action [26].

Greater internal motivation is associated with more interest, engagement, and positive coping with failure and—in the realm of health care—with greater adherence to medications and better long-term health outcomes [26]. Motivation is likely to become more self-determined when the needs for competence, relatedness, and autonomy are fulfilled. The notion of need fulfilment leading to internalization of motivation was a guiding theme in our analysis.

#### Taking and Relinquishing Control: An Alternative View on Patient Empowerment

It is widely assumed that self-efficacy, mastery, and control are outcomes of patient empowerment [5] and that a patient’s being or feeling in control of a disease is beneficial for treatment [28-31]. This view of patient empowerment focuses on “activating” patients who, as a result of “rejecting the passivity of sick role behavior and assuming responsibility for their care (...), are more knowledgeable about, satisfied with, and committed to their treatment regimens” [32]. Indeed, Salmon and Hall [7] argue that “The validity of the view that patients should be empowered to take control and make choices is...widely assumed to be unassailable.”

Aujoulat et al [6] have questioned this common model of self-efficacy and bodily control. They argue that this view ignores the patient’s need for security, self-determination, and a continuous sense of self. They present a concept of patient empowerment characterized by two processes: first, patients need to separate their illness from their selves by taking control and, second, they need to accept their illness and illness-driven boundaries by relinquishing control. *Taking control* involves learning to control the disease, developing cognitive coping strategies, controlling social roles, and separating the disease from one’s own identity. Psychological research [33-35] confirms that this process helps chronically ill people to regard themselves as fundamentally sound and healthy. *Relinquishing control* involves asking for help, accepting that not everything can be controlled, and developing a sense of coherence, as well as awareness and acknowledgement of personal boundaries. In the study by Aujoulat et al [6], participants explored the origin or cause of their illness, for example, genetic predisposition and precipitating factors, as part of the process of relinquishing control. Based on this work, we discuss to what extent it is reasonable to push patients to take more responsibility and control with PCEHRs.

## Methods

As noted earlier, our initial aim was to better understand the lived experiences of families using a PCEHR. The focus evolved toward how the PCEHR supported or obstructed people’s sense of being in control (as described in more detail in the following section) as the study progressed. A qualitative methodology suited the research questions being addressed, as it focuses on patients’ experiences and practices [36]. Before the study commenced, ethical clearance was obtained from a UK National Health Service Research Ethics Committee (reference number 14/NS/0045).

### The System

**Figure 1:**
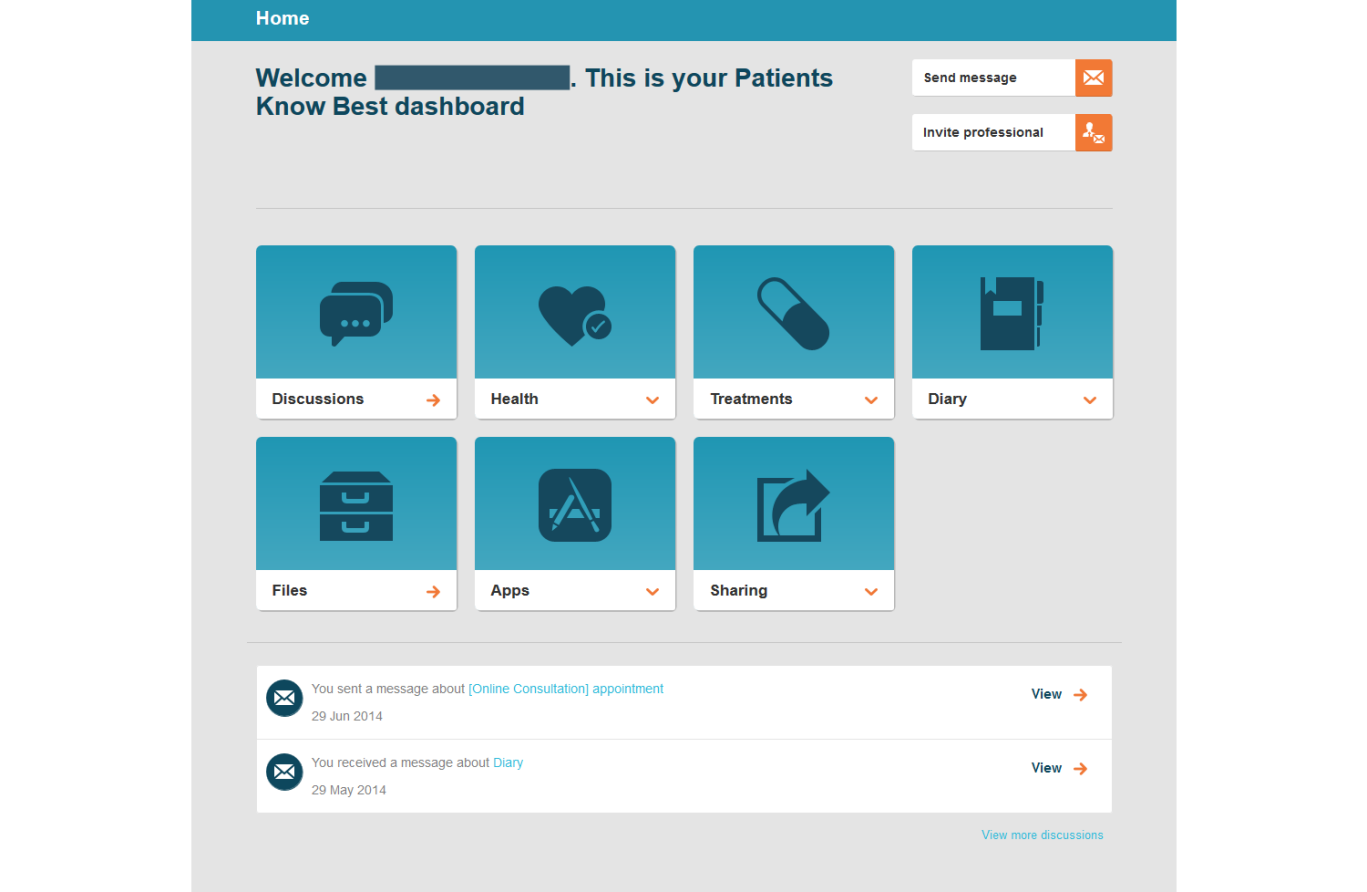
Screenshot of the patient interface of Patients Know Best.

The PCEHR used in this study was Patients Know Best [37] (Figure 1). It allows patients and clinicians alike to upload, enter, view, and edit various health data (eg, symptoms, medications, diagnoses, test results, and body measurements). Changes are tracked and previous versions can be retrieved to ensure that both clinicians and patients can use the record at their convenience. In addition, it provides features that are traditionally not part of a health record, such as electronic messaging, video conferencing, and file management. Although the PCEHR can be tethered to the EHR of a hospital, this was not the case in our study setting. Therefore, the PCEHR contained information and documents that members of the clinical team, patients, or other doctors involved in the patient’s care uploaded.

### Context and Participants

Data gathering took place in 2 departments within 1 hospital; Department A specializes in intestinal failure and Department B in inflammatory bowel disease.

Department A cared for around 20-30 highly complex outpatients between the ages of 1 and 25. These patients were dependent on parenteral/intravenous nutrition (PN) that was managed at home by their parents. Their parent/s had undergone formal training that taught them how to safely administer PN. This requires the patients to take a lot of responsibility and control as soon as they became outpatients. The PCEHR had been introduced about 2 years prior to the start of this study, and many families had been attending Department A for years before that. Because of their medical complexity, many patients were in the care of multiple medical teams. To coordinate care with the medical teams near patients’ homes, and to provide the patient families with the necessary support in between 3- or 6-month consultations, the hospital team had frequent contact with them via telephone calls and via the PCEHR.

Department B cared for 60-80 children and teenagers under the age of 18. The treatment of Department B’s patients usually consisted of taking prescribed medication, depending on the severity of symptoms, and patients were often required to adhere to a special diet. In Department B, nurses and a few patient families had been using the PCEHR for about half a year when the study commenced. Because of the large number of patients, consultants did not use the PCEHR. According to one of the clinicians interviewed, due to the slow consent and sign-up process and a low take-up rate, only about 10% of patient families in Department B were using the PCEHR when the study was conducted. Consequently, it was only possible to recruit 2 participants from Department B.

We interviewed all patient families of Department A who signed up for the PCEHR and agreed to participate in our study (in total 14 patient families) and 2 patient families of Department B to get insights into whether our results generalize across departments. We also interviewed 7 clinicians from Department A and 4 from Department B to gather their complementary perspectives on families’ experiences.

**Figure 2:**
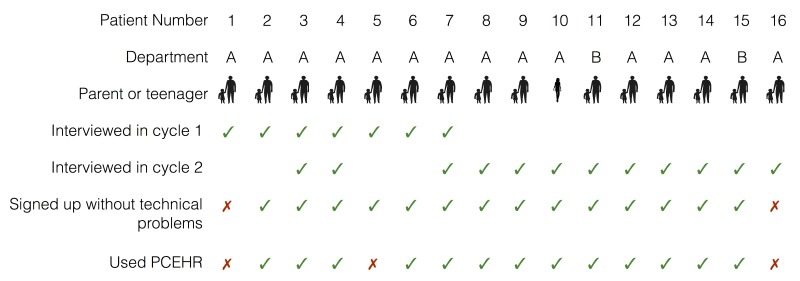
Overview of interviewed patient families.

Figure 2 summarizes participant profiles of patient families, from both departments. In the following, we refer to patient families as P1 to P16 and to clinicians as C1 to C11. Participant numbers were assigned sequentially with P1 being the first and P16 being the last patient family participating. In 15 of the patient families, a parent of the patient was the principal participant; P10 was a teenage patient who participated directly.

Members of the clinical team were invited to take part by the researcher; patient families suitable for the study were identified, invited to take part, and consented by hospital staff.

### Data Gathering

The data gathering comprised a total of 23 hours of observations and approximately 9 hours of interviews. Interviews took between 7 and 45 minutes (with an average of 23 minutes). Our interviews aimed at understanding participants’ use and nonuse of certain features of the PCEHR and their underlying motives.

Our study was structured in 2 cycles of exploratory data gathering and data analysis. Some participants were interviewed in both cycles (Figure 2). In Cycle 1, study participants were recruited with the goal to get as broad a representation of users of the PCEHR as possible (including patients who use the PCEHR themselves, carers who use it on behalf of a patient, and various people involved in a patient’s care, eg, nurses, consultants, clinical assistants, dieticians, and pharmacists). Following a critical incident technique [38], in this cycle of data gathering and analysis, we asked about specific events involving the PCEHR rather than for general opinions, which tend to be less accurate. The interview script included questions such as the following:

- When was the last time you used the PCEHR?
- Do you remember a time when using the PCEHR helped you a lot/frustrated you?
- Can you walk me through how you used it?
- What was your goal?
- Do you use the PCEHR for other purposes as well?

We also included open questions that prompted participants to reflect on any changes caused by the PCEHR, such as the following:

- Do you feel the PCEHR has changed the relationship between you and your clinicians?
- Do you feel the PCEHR helps you to make better decisions in your/your child’s care?

In the second cycle of data gathering and analysis, having identified important dimensions of variability in our data (such as motivation to use PCEHR and to take responsibility and control in the treatment), we adopted a theoretical sampling approach: we recruited new participants who we anticipated might show new or extreme manifestations of our identified dimensions, such as very intense PCEHR use. In this cycle of data gathering and analysis, we specifically asked questions referring to the identified dimensions of variability in the data. For example, we asked participants what features of the PCEHR they used or would use, to classify their motivation to use PCEHR.

Most interviews were conducted during 9 hospital visits; 3 took place by telephone. In practice, several factors constrained data gathering with patient families. First, only outpatients had been invited to use the PCEHR, and they visited the hospital infrequently; where possible, we scheduled interviews with PCEHR users to coincide with visits. However, later in the process, we conducted phone interviews as well. Second, patients’ families live extremely stressful lives, and had limited time to spare after their appointments. Many families came with several small children, had to visit several departments in the hospital, and had many tasks to juggle during their visits. As a consequence, some interviews were rushed. A total of 2 interviews took place while walking with a family from one part of the hospital to another, making audio recordings impracticable. In these cases, handwritten notes were taken. This practice is less than ideal, but it was a necessary adaptation to the constraints of the hospital setting [39,40], and meant that participants were not excluded simply because they did not have time to participate in a more formal interview.

Observations focused on how the clinical team made use of the PCEHR and other tools during consultations as well as on patient-clinician interactions in general. Interviews and observations with clinicians proved more straightforward to plan and conduct than those with patients and their families.

### Data Analysis

Within both cycles of data gathering and analysis, we adopted a Grounded Theory approach, as defined by Strauss and Corbin [41]. The first stage, “open coding,” involved deconstructing transcript data and field notes into short phrases capturing key components of the participants’ perceived reality. The second stage, “axial coding,” involved comparing these text fragments within and across participants’ datasets, resulting in loose concepts and ideas. The third stage, “selective coding,” aimed at identifying the relationships between these ideas, resulting in a structured framework of higher-level themes. In the final step, “theoretic coding,” these themes were compared with existing theories in the literature, an integral part of Grounded Theory [42].

**Figure 3:**
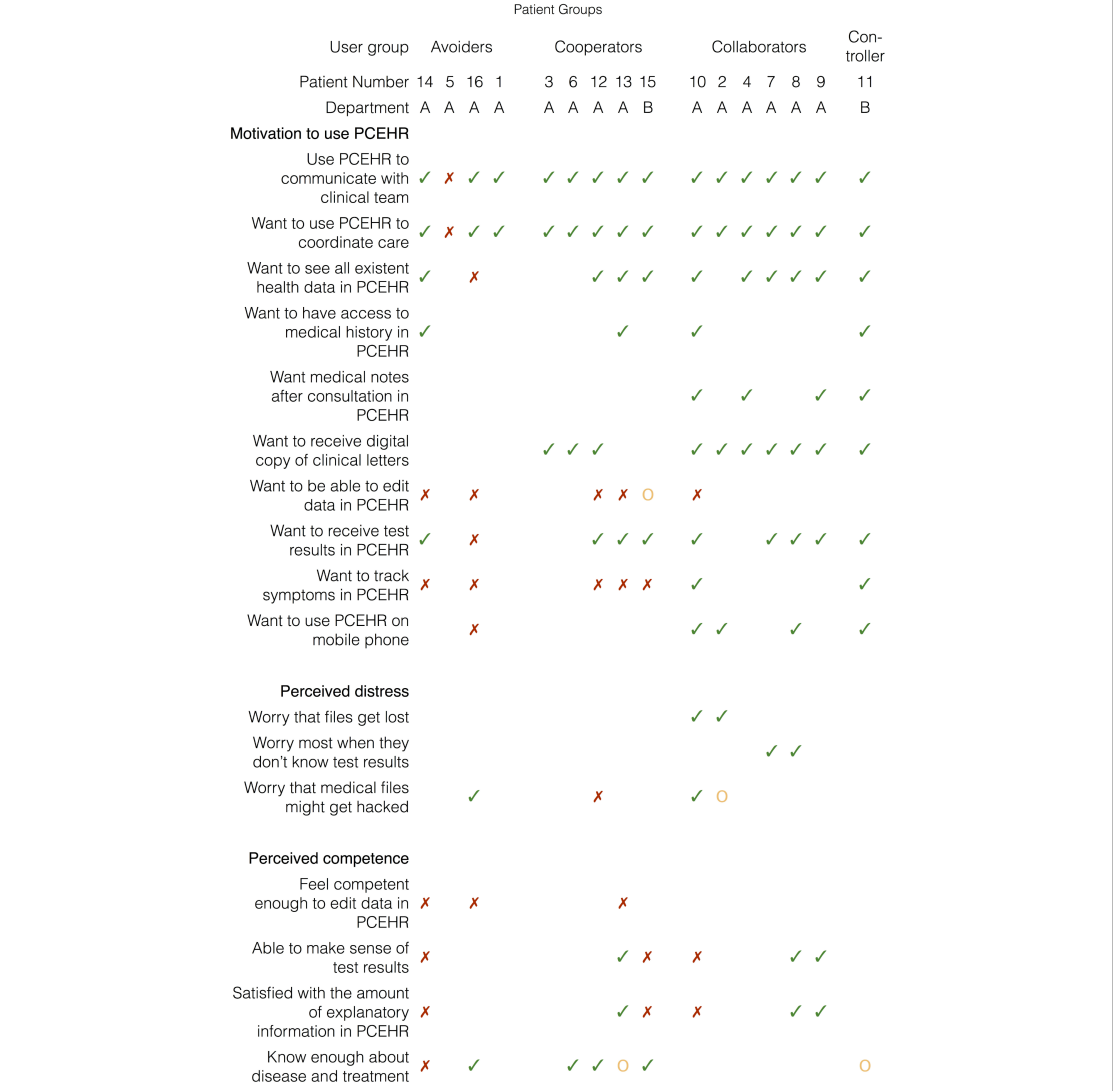
Grouping of participants and reported needs and practices.

Earlier transcripts were recoded, and early participants were asked to participate in follow-up interviews until newly gathered data ceased to generate new codes, a stage termed “conceptual saturation” [43]. The codes identified from participants’ stated attitudes and needs are summarized in Figure 3. In this figure, participants are ordered according to subsequent stages of analysis as presented in the following section.

For every participant and code, we assigned one of the 4 variables: “need felt” (indicated by a green tick in 5), “partly/unsure” (yellow circle), “need not felt” (red cross), or “no statement” (indicated by the absence of any mark). To assign these values, we read through all participant transcripts again and identified statements that revealed the participant’s attitude toward a code.

We assigned the value “need felt,” when the participant mentioned a need either spontaneously or (especially in later interviews) when asked by the researcher—for example, “Do you use the function to track symptoms in the PCEHR? Why (not)?”.

We assigned “no statement” when the interview did not reveal a participant’s attitude toward a need at all. Both the openness of participants and the time frame and context of the interviews (described earlier) influenced whether the participant made a statement about a need or not. In later interviews, we asked more theoretically guided questions, resulting in more statements on identified needs.

We assigned the value “unsure” when a participant was asked about a specific need and was not sure about the answer, for example,

Would you like to be able to edit data?

Interviewer

I don’t know.

P15

or when the participant’s answer was ambiguous, for example,

Are you interested in participating more in medical decisions?

Interviewer

Well, I noticed, that I have faith that these people know more than me, that they are educated. There would always be the case when I trust the decisions that they make. If I wasn’t happy or if it wasn’t the best for my child, ultimately the decision is still down to me. But I have to trust them, I have to.

P14

Based on the codes in Figure 3, which were shaped by both the data analysis and the theoretical perspectives presented earlier, we identified 8 dimensions of variability, as outlined in the following section. These dimensions of variability are not mutually exclusive. The dimension “motivation to use the PCEHR” is conceptually different from the other 7 dimensions (it is what we set out to examine), while the other dimensions emerged from our data and seemed to correlate with PCEHR use. While previous work suggested that computer, reading, or health literacy influence the adoption or nonadoption of a PHR [44], in our data the patient’s adjustment toward the disease more clearly correlated with motivation to use the PCEHR.

#### Motivation to Use the PCEHR

Motivation to use the PCEHR guided the grouping of our participants into a controller, collaborators, cooperators, and avoiders. This dimension was directly inferred from our data by statements on how often and how many features of the PCEHR participants used or wanted to use (eg, some participants used the PCEHR to track symptoms, medications, and other treatment-related markers, whereas others did not). Moreover, some participants’ feature requests indicated that they would use the PCEHR even more if possible (eg, to see all existing health data, have access to the medical history, receive medical notes after a consultation, receive digital copies of clinical letters, edit data, receive test results, and use all features of the PCEHR on a mobile phone).

#### Continuum From Avoidance- To Approach-Oriented Coping

Motivation to use the PCEHR correlated with participants’ coping styles. As detailed earlier, coping styles vary from approach oriented to avoidance oriented. Statements that indicated an approach-oriented coping style included the following: “Investigating the data is a way of coping for me,” “I use the PCEHR to prepare for clinical appointments,” “I want to understand medical decisions,” and “I want to take part in medical decisions.” Statements that indicated an avoidance-oriented coping style included “I try to avoid thinking about the disease,” but avoidance was typically characterized by the absence of more approach-oriented statements.

#### Continuum From Amotivation to Internal Motivation

4 dimensions of variability were derived from the SDT [26]: perceived competence, perceived autonomy, perceived relatedness, and (hence) internalization of motivation. During analysis, the first 3 were inferred from our data, whereas the last was inferred from the other 3.

Statements that indicated perceived competence included “I feel competent enough to edit medical record,” “I feel I’m able to make sense of the test results,” “I’m satisfied with the amount of explanatory information in PCEHR,” and “I feel I know enough about the disease and the treatment.”

Statements that indicated perceived autonomy included “I double-check all medication prescriptions,” and “I want to be able to edit data in the PCEHR.”

Statements that indicated perceived relatedness to health care professionals included “I completely trust my doctors.”

Statements that indicated intrinsic motivation included “I want to understand medical decisions,” “Investigating the data is a way of coping for me,” “I double-check all medication prescriptions,” and “I use the PCEHR to prepare for clinical appointments.”

#### Control Taken and Relinquished

The last 2 dimensions were derived from the work of Aujoulat et al [6], namely, the amount of control taken and control relinquished. The amount of control taken and control relinquished were both directly inferred from our data. The use of features such as feature tracking, the investigation of test result, and the double-checking of medication prescriptions indicated a high amount of control taken, whereas statements such as “I trust the decisions of my doctors” and “I rather spend quality time with my child than to think about the condition” indicated a high amount of control relinquished.

We clustered participants into groups, based on the codes in Figure 3. We refer to the outlier as controlling, and the groups as collaborating, cooperating, and avoiding.

To validate our classification, we conducted a 2-step cluster analysis in SPSS using 4 clusters and all codes. The results of the cluster analysis matched the clustering based on the researchers’ judgment for 12 of the 16 participants. We resolved the discrepancy of the 4 exceptions [P14, P3, P6, and P10] based on their location on the identified dimensions.

We assigned P10 to the collaborating group, even though the cluster analysis assigned P10 as a controller. Unlike P10, P11 was preoccupied with tracking symptoms, medications, food intake, and other disease markers. The *low relatedness* that characterized P11’s behavior was not observable in P10 either.

We assigned P3 and P6 to the cooperating group, whereas the 2-step cluster analysis assigned them to the collaborating group. As these interviews took place in Cycle 1, data were thinner than the data we had about other participants. The key reason for our assignment decision was that P3 and P6 said they wanted to spend as little time as possible with the treatment or the PCEHR, either because they were stressed [P6] or because they prioritized spending quality time with their children [P3]. They shared this characteristic with other patient families in the collaborating group who used the PCEHR mainly to communicate with the clinical team and to coordinate care efficiently.

Finally, we assigned P14 to the avoiding group, whereas the cluster analysis assigned this patient family to the cooperating group. Patient families in the cooperating group seemed to have accepted the illness and consciously relinquished control, whereas P14 leaned toward denial. As a result, this patient’s family spent as little time with the PCEHR as possible. What differentiated P14 from other participants in the avoiding group was that this family had used the PCEHR, whereas other participants in the avoiding group had not.

## Results

Some PCEHR user needs were common to all participants in our study: the need for quick and easy communication with the clinical team; to coordinate care efficiently across multiple medical teams; to conceptually understand the PCEHR and receive adequate training; and to access the PCEHR on mobile devices. However, we also found differences between the PCEHR needs of patient families based on their motivation to take responsibility and control of their health management. As described earlier, 3 groups of users and an outlier were identified. The following sections elaborate on the behaviors, attitudes, and needs of these groups, starting with the outlier with the most intense PCEHR use and moving to the group with least use.

### The Controller

One patient family (P11) used the PCEHR much more than all other participants. We present this patient family as a singleton because we believe that this observation might be of interest for researchers who observe similar behavior and because they have important properties when it comes to designing and deploying a PCEHR. We cannot be sure how common such behavior is, and therefore, these results should be interpreted with caution.

This patient family (P11) reported negative experiences with health care providers, and thus, learned to take an unusual degree of control of their child’s treatment, in an attempt to ensure and improve the quality of care. As a result, P11 used the PCEHR extensively as a personal tool for health management, exemplifying their approach-oriented coping style,

We signed up for it and also invited a number of consultants who are connected with [my child’s] health. I used the symptoms charts; I put lots of notes on there; I’ve put medicines on there; I put everything on there. It’s more of a record for me as a parent so I can go back to this. But I thought in the beginning I would be able to use it as a tool when I speak to clinicians, to have it on my phone as an app to get the information, but I found that of all the clinicians I invited, only this hospital and the local podiatrist signed up for it, no one else has done it, I’ve chased it a number of times, [my child] sees about 12 different people.

P11

The aforementioned statement illustrates that the *perceived relatedness* of this family was low. When the family received blood test results during consultations or as PDF attachment to a message, they wished to store this information in the intended section of the PCEHR. However, P11 felt insecure about entering these data themselves, indicating low *perceived competence*:

Initially, I wasn’t sure I was doing it right. I’ve actually uploaded blood test results myself, even though I don’t feel comfortable doing it, because there are lots of [differences] between the labs that are doing [measurements], but I uploaded some.

P11

According to SDT, low *perceived competence* and *relatedness* impede the development of *intrinsic motivation*. Indeed, P11’s motivation to take control and responsibility seemed to be at least partly driven by fear and mistrust. Although approach-oriented coping is consistently associated with indicators of positive adjustment to chronic disease [20], this patient’s family experienced *high distress:* The family members were worried about whether they were using the PCEHR correctly (see above) and whether the care received from the medical teams was good:

The communication [with the local surgery] is really poor. If for example my medication has changed here, by the time it gets to the surgery, it takes them about a week, 4-5 days, to actually process the fax that has come through to them to get into the system. And I had a case where they’ve duplicated the medication; they’ve done it wrong, so every time I’ve got to check and double-check what they are issuing.

P11

**Figure 4:**
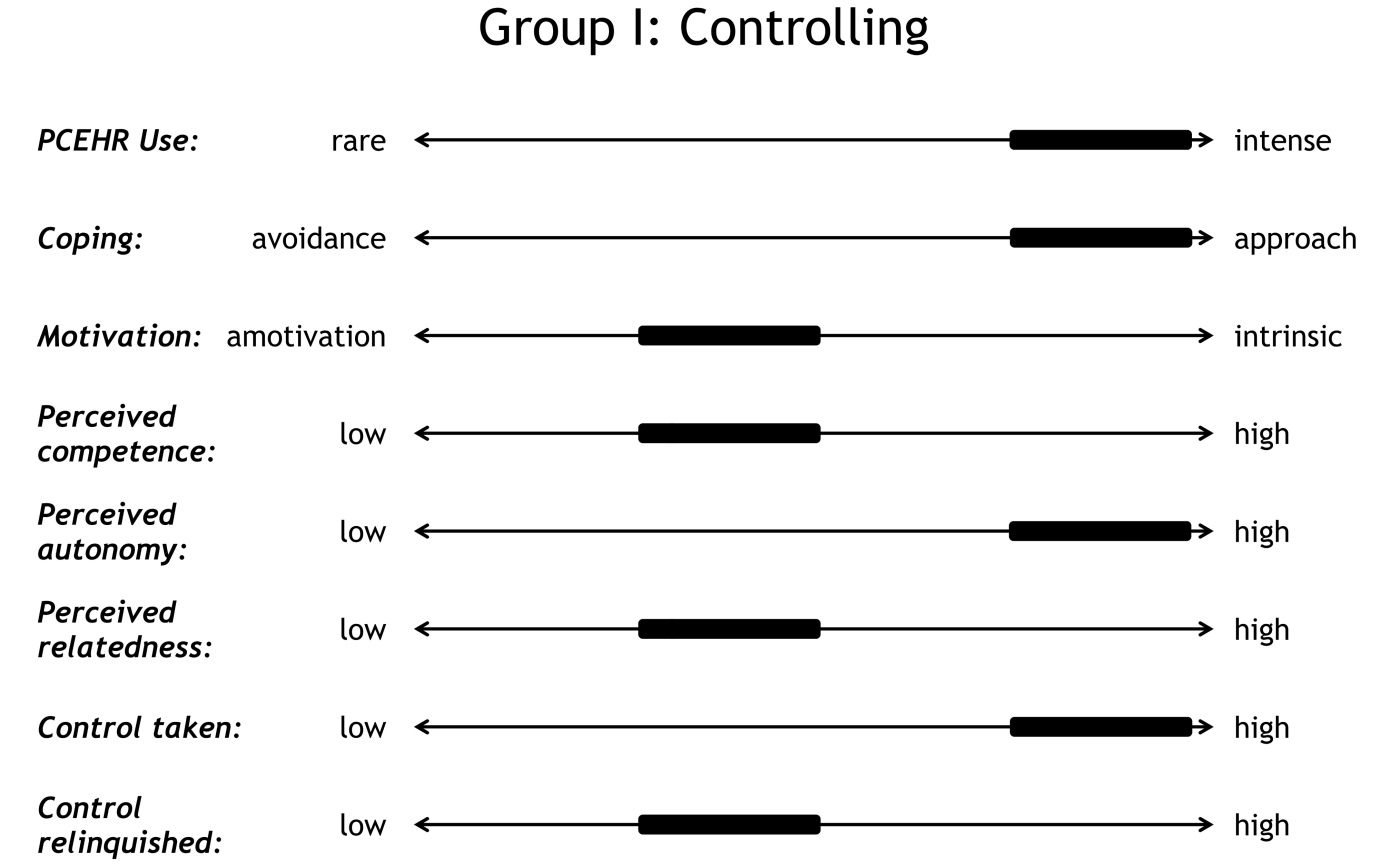
Profile of the controller.

This patient’s family demonstrated that *intense PCEHR use* does not necessarily correlate with successful coping. Therefore, P11 exemplified that taking a *high amount of control* does not necessarily indicate patient empowerment (as stated by Aujoulat et al [6]): the complementary process of relinquishing control was not reflected in P11’s statements. These features are summarized in Figure 4.

### The Collaborators

**Figure 5:**
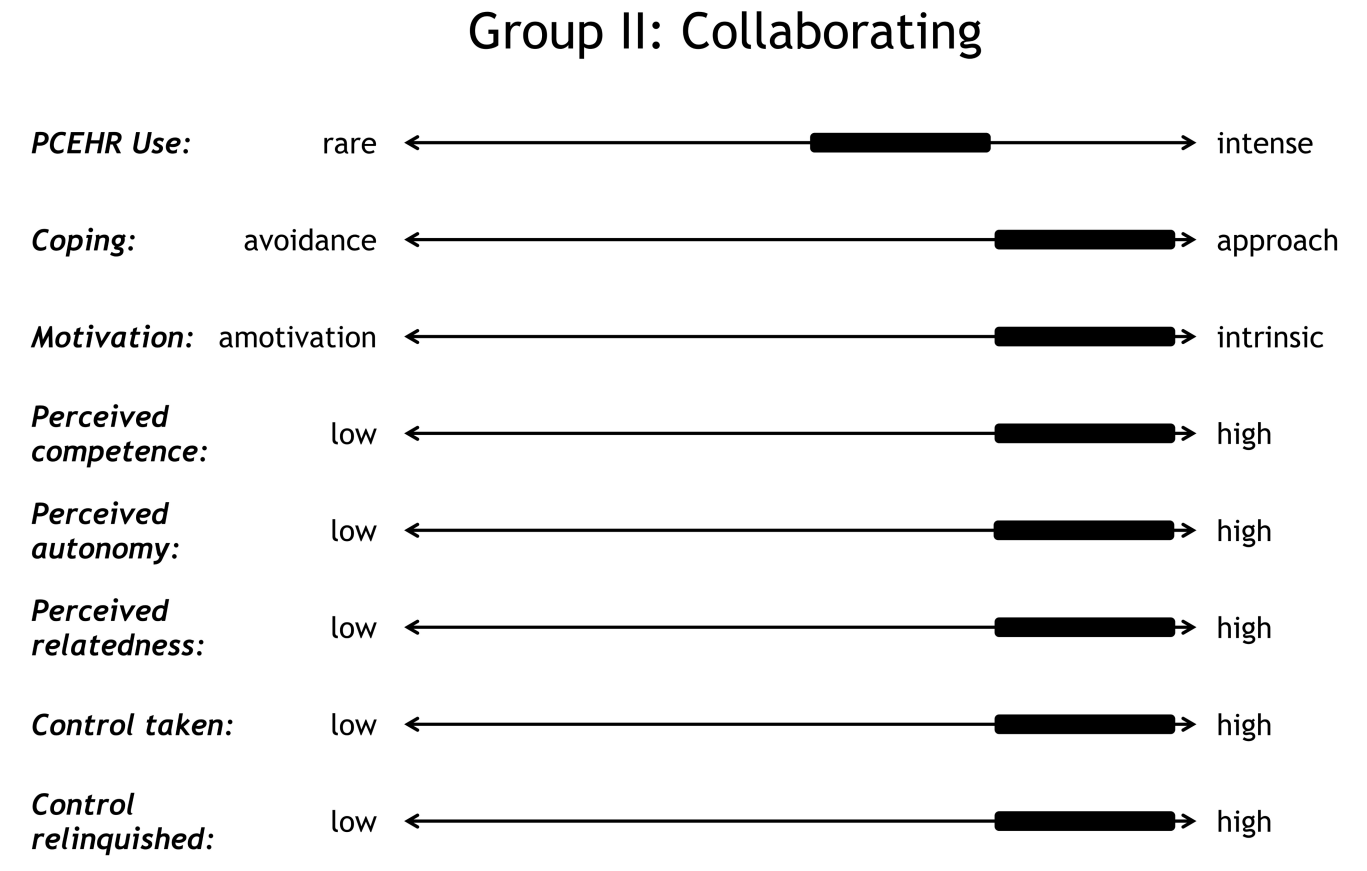
Profile of collaborators.

The collaborating group (P2, P7, P8, P9, P4, and P10) shares one characteristic with the controller: proactivity. However, what differentiates the collaborators from the controllers is that they perceived high levels of *competence*, *autonomy*, and *relatedness*. Thus, their proactivity is grounded in *intrinsic motivation* (Figure 5). In contrast to the controller, the collaborators experienced *low levels of distress* although both of them adopted an *approach-oriented coping style*. Moreover, the collaborators perceived high treatment-related *competence*, as illustrated by the following statements:

My GP knows that I’m much more of an expert than he is and he believes me with all I say about my child’s care.

P9

Their *autonomy*, or internal locus of control, was accompanied by an awareness of the limitations of their clinical team:

There are a lot of times when parents by going back over the results find something and then ask the consultant. Because they’ve got soooo many patients, they won’t have the time to do what you are doing. When you are stressing about your child, you know what I mean.

P8

However, this awareness did not negatively impact on the families’ confidence in the clinical team. Instead, they developed motivation to ensure that nothing was missed in the care of their child. They, therefore, collaborated with the clinical team, indicating their *perceived relatedness*. P9, for example, described how they discussed doubts, suggestions, and decisions together.

Recently I requested to see [a specific test] result before clinic. This week it was really important because looking at the result on Monday I saw something unexpected and asked my consultant through [the PCEHR]. She then decided to do a [specific] X-ray when we came in on Tuesday...The results of that one could now mean that my child needs to have a surgery. So it made quite a significant difference that I asked for the test results before clinic. I also pointed out that there was an X-ray in December and I reminded them that this ought to be compared to the new one that was made.

P9

P9’s clinician reported the same incident and was happy about P9’s involvement:

Recently actually, I had a little lad, it was part of his annual review, there were several investigations and that involved a chest X-Ray. And the little lad had some [symptoms]. So the mother questioned that and I hadn’t actually seen the film myself yet. And as it turned out he needed [a specialist] X-ray and is now most likely going to end up with surgery. So that was very helpful the mother chased it up herself. He was very well when he came into clinic so there wouldn’t have been any immediate concern about this. So it is helpful in that respect. I think it gives parents a little bit of responsibility as well which I think is the whole idea of the new NHS that patients are actually responsible for their own health, to an extent.

C6

Given the *competence*, *autonomy*, and *relatedness* these families experienced, their *high intrinsic motivation* to use the PCEHR and to take control and responsibility in their care is consistent with SDT. In a partnership with their clinicians, patient families were able to both take and relinquish control, and experienced *less distress* than the controllers (P8 and P9 worried most when waiting for test results, see the following conversation between the interviewer and P8). We, therefore, perceived the collaborators as empowered, according to the definition of Aujoulat et al [6]. Indeed, participants themselves reported that the PCEHR helped them to cope with their situation by giving them the means to investigate the medical data themselves. P8, for example, reported that seeing if they could find something the doctors were missing helped them to cope with the situation:

What does that mean to you to get the results immediately?

Interviewer

Less worry because every parent sits there and wonders and I know a lot of friends who use [the PCEHR]. And you sit and you worry about what the result is and so to get them quicker puts you out of your misery, if you know what I mean, you got to relax much, much easier. [...] I think that’s the way I cope with it. I have to see if I can find something out that they are missing.

P8

Clinicians confirmed that the way these patient families used the PCEHR was helpful and desirable,

The families use it very appropriately. Sometimes excellent actually, [...] when they actually upload a picture then you say “ah yeah it’s this or that”, then you can say “this is ok, I’m not worried” or “this is not ok you should see your local hospital” or “we actually want to see you here”. I think that is quite useful.

C6

As the way the collaborators used the PCEHR helped both patients and clinicians, we infer that the current design of the technology addresses their needs well. However, it is less suited for cooperators and avoiders.

### The Cooperators

**Figure 6:**
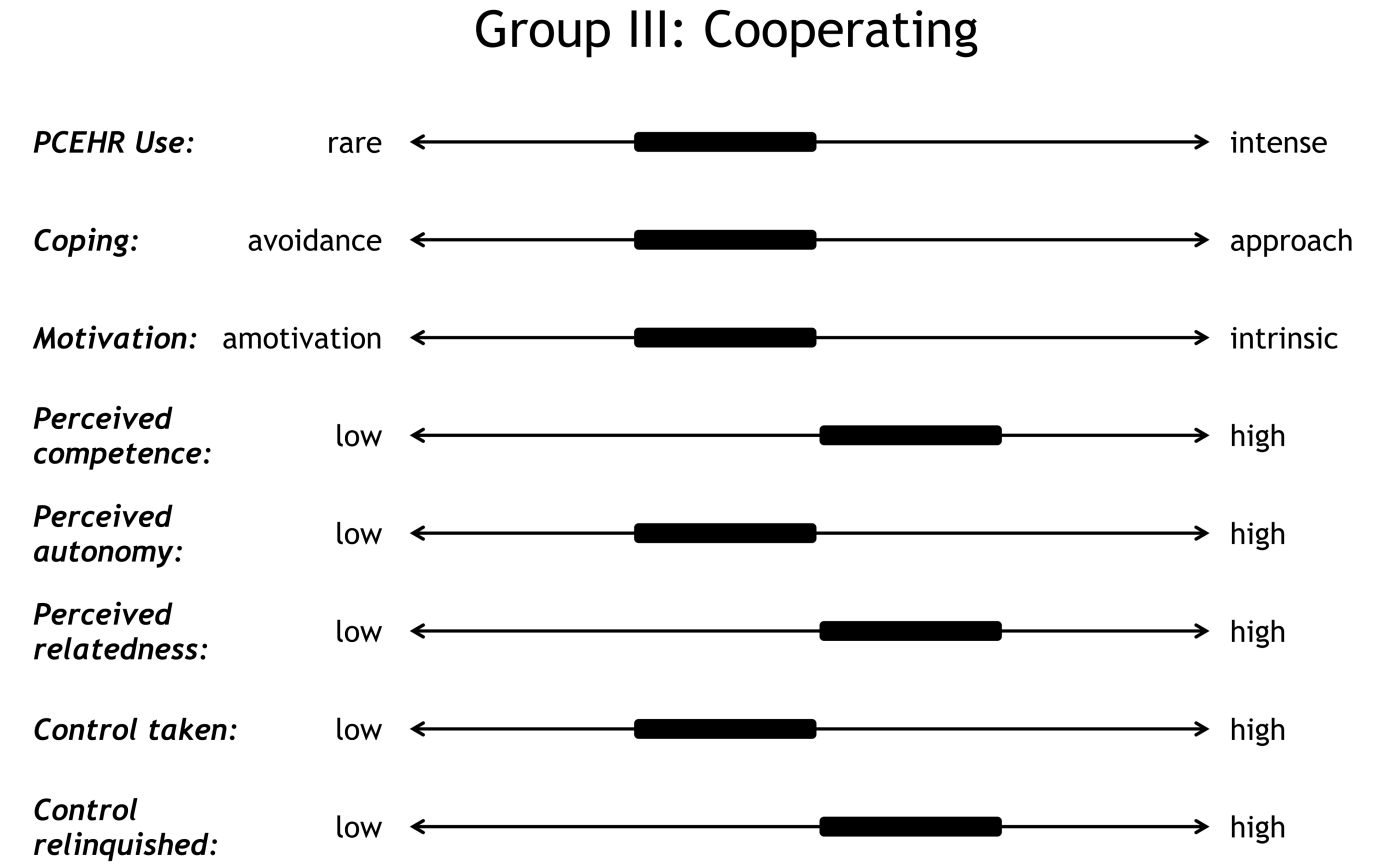
Profile of cooperators.

The 5 patient families in the cooperating group (P3, P6, P12, P13, and P15) displayed *competence*; for example, P6, P12, and P15 said they felt like they knew enough about the disease and the treatment and P13 was confident in interpreting test results. They also displayed *relatedness* (eg, P6, P12, P13, and P5 said they felt well supported by the clinical team and trusted their doctors’ decisions), like the collaborators. However, the cooperators did not want to use the PCEHR as much as the collaborators. They were equally interested in having access to health data, receiving blood test results immediately, and receiving a digital copy of clinical letters via the PCEHR, but mainly to make managing the condition more efficient and to reduce the time and effort they needed to spend on it. In contrast to the collaborators, the cooperators were not interested in additional features of the PCEHR, such as symptom tracking and journaling, and they *used the PCEHR less intensely* (Figure 6):

Do you think tracking symptoms might be useful for you?

Interviewer

No, I don’t think so... I don’t know if it could be too much. You’d sit there thinking, which symptoms and worry. Like when you Google things you get a headache, don’t you? “Oh god, oh god, it’s this.” It would be like that.

P13

When being asked about the reason for this, many of them replied that they did not want to think about the condition when not really necessary and they would rather spend quality time with their children. While “seeing if I can find something the doctors might have missed” [P8] was a coping strategy for the collaborators, the cooperators tried to think as little about the condition as possible (Figure 6).

As cooperators perceived a similar level of *competence* and *relatedness* to the collaborators, *autonomy* is the only factor that could have impeded the development of intrinsic motivation according to SDT [45]. When being asked if they do or do not want to take part in medical decisions, P12 replied yes, P13 was not sure, and P15 did not want to take part:

Would you like to participate in medical decisions?

Interviewer

No, up to the doctor…

P15

Would you like to participate more in the care of your child?

Interviewer

No I rather want to live life without thinking about it too much. I know the doctors are very busy and I am also very busy with these two children.

P15

We also noted that the cooperators did not display the same awareness of their doctors’ limitations as the collaborators.

On the continuum from *approach to avoidance*, the cooperators adopted a slightly more avoidant coping strategy. They consciously decided to relinquish a certain amount of control and were often articulate about their reasons, for example, that they preferred not to think about the illness (see statement above). As, according to Aujoulat et al [6], relinquishing control is equally important for patient empowerment as taking control, the cooperators can also be regarded as empowered. As the cooperators did not show any sign of anxiety or distress, there is no obvious reason to encourage them to use more PCEHR features unless health care providers expect clear benefits in terms of treatment outcome. A PCEHR that reflects this insight would not nudge patients to make use of all features but allow them to spend just the necessary amount of time to keep track of key data.

### The Avoiders

**Figure 7:**
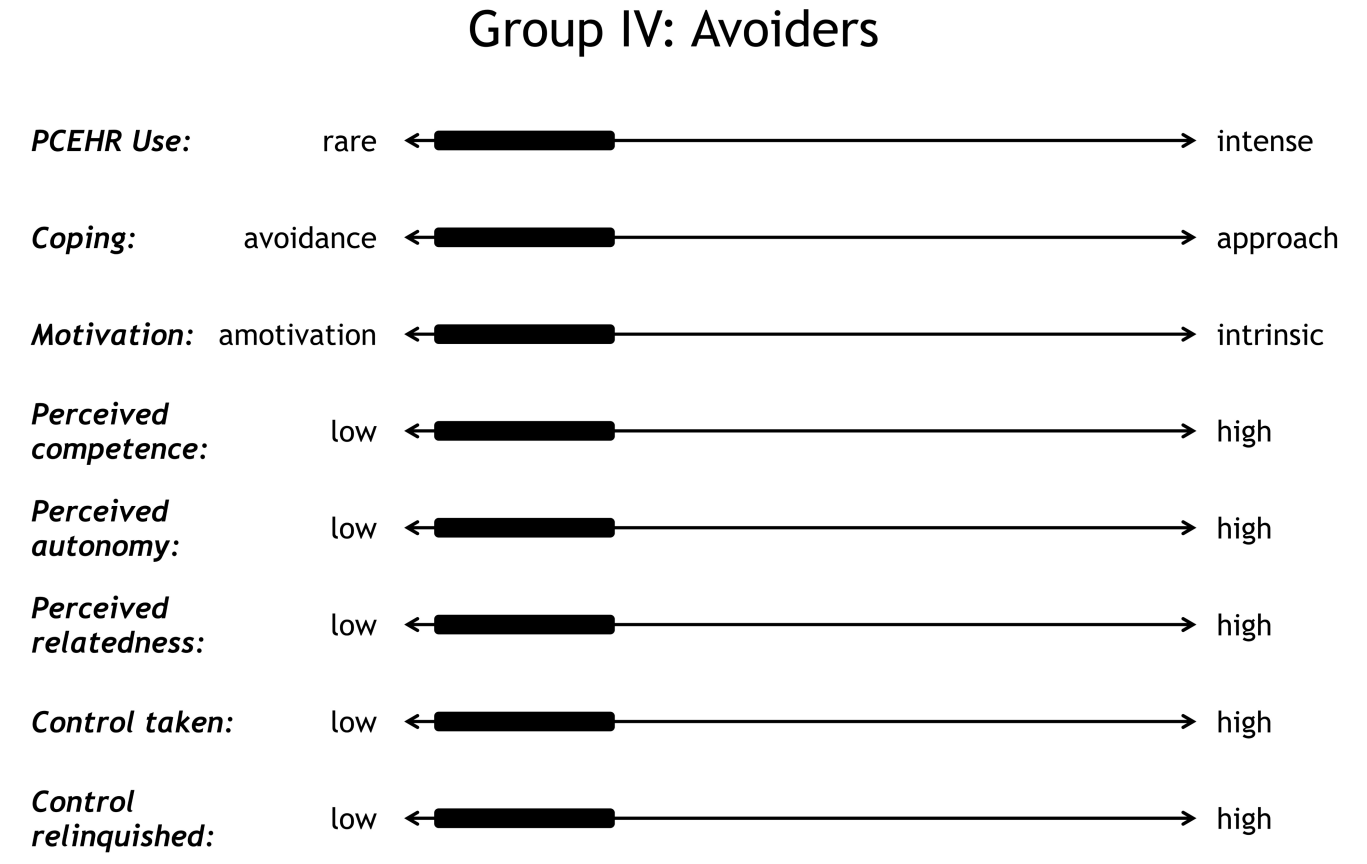
Profile of avoiders.

The avoiding group [P1, P5, P14, and P16] is characterized by engaging neither with the PCEHR nor with the disease and by a tendency toward denial (Figure 7). In this study, there were no participants who were identified as engaging well with clinicians and treatment while actively avoiding using the PCEHR. It is possible that in a larger study such people might have been identified, but we speculate that the benefits that the PCEHR provided for families would mean that those who were actively engaged in managing their child’s health would be motivated to learn the basic features of the PCEHR.

Like cooperators, P14 and P5’s priority was to minimize the impact of the illness on their lives. However, the cooperators seemed to have accepted the illness and decided to live lives that were as fulfilled as possible. Avoiders by contrast leaned toward denial. As a result, P14, for example, spent as little time with the PCEHR as possible:

I try to normalize life. I escape the treatment, that’s what I do 12 hours a day. On the day when [my child] doesn’t have to be on the machine, I pack all things away and just ignore it. Maybe that’s why I don’t spend so much time on the PCEHR. I don’t know if it helps to deal this way with it but when you can’t deal with it I don’t like to wallow in it.

P14

P5 had never used the PCEHR because this family was overloaded with work and the care of their chronically ill child:

I have a work. So at the time I come home from work I get TPN [total parental nutrition] out of the fridge so that it is at the temperature to put [my child] up. Then I go and collect my children from the childcare, then I get home and I make two different types of dinner due to [my child’s] special requirements and then I put my [child] on the TPN, then I put [my other child] to bed, then there is time to clear up and sit down. Yeah, there is no time. I get the bags ready for the next day for school for [my children] and me to go to work...I’m exhausted; I’m so sleepy.

P5

The remaining 2 patient families [P1 and P16] in the avoiding group had never used the PCEHR because they had experienced technical problems when initially signing up, and had not pursued gaining access.

Avoiders did not feel that they understood medical data and decisions, unlike patient families in the other groups. While P16 preferred to wait for the next clinic appointment in 3 or 6 months, so that blood results would be explained properly, P14 was interested in receiving them but needed more explanatory information to make sense of the data:

Well, I don’t know what [the blood results] mean anyway, so I always presume that they’re normal, and if they weren’t I imagine that someone would tell me. I don’t really know what I’m reading; I don’t really know what any of it means.

P14

Would you like to get more information with it to help you understand what they mean?

Interviewer

Yes. You just get the figures at the end, which means nothing to me.

P14

P14 and P16 were not confident in their own *disease-related competence* and, consequently, did not prefer to annotate their medical record:

Would you like to be able to comment on medical notes? Maybe there would be something that doesn’t appear in the medical record but you think it might be important.

Interviewer

No because sometimes, I don’t know what is important and what isn’t when doctors talk to me. So, it’s the same kind of thing like with the blood test results. I always kind of presume that if it is important than it would be written down. And if it is something that is just mentioned and it’s not in the notes I would imagine that it doesn’t need to be. You know it’s just impossible for them to put down absolutely everything.

P14

Although P14 expressed trust in the competence of their doctors, they did not always feel well supported by the health care system:

When we came here four months ago, it took me a while to figure out how things worked at my local, and here that was very confusing and frustrating for the first few months [...]. And the trouble really was, there is nothing worse than a consultant standing in front of you and saying that he doesn’t really know how to look after [your child] and then getting another one saying it. You know, all these doctors are saying that they have no experience at all with children like mine; they just haven’t got the knowledge. And I’m also acutely aware that we cannot come back to this hospital where the experience is.

P14

This lack of *relatedness* and the lack *of perceived competence* could have inhibited the development of intrinsic motivation to use the PCEHR and to take more control and responsibility in the treatment, according to SDT [26]. As a result, the patient families neither took nor consciously decided to relinquish control in the way necessary for patient empowerment. Therefore, the avoiding group was not empowered, according to Aujoulat et al [6].

In contrast to the cooperators, the avoiders experienced anxiety and distress: P14, for example, was distressed because they did not feel that they understood diagnosis and test results enough to comprehend the doctors’ conclusions:

[...] I’m not sure now that after the surgery these results still stand. And when they are talking about opening my child’s diet I’m kind of concerned that opening the diet...that these test results don’t still stand because things have changed since then. I would like to be able to educate myself because that would help me to make day to day decisions with confidence rather than guessing. So I think having access [to all the data] would be very helpful. If I would have more information I would feel like I can make informed decisions.

P14

While access to more health data and information would have helped P14, other patient families experienced more distress when confronted with such data. P8, for example, mentioned that a friend does not want to see test results in the PCEHR, because it is too upsetting:

A lot of people worry, I have a friend who doesn’t want to see it because it is too upsetting, so would rather not look through the results. But for me, I need to know what’s happening. I’d rather be above it before something is happening and not know.

P8

Similarly, a clinician (C2) mentioned that some patient families want to see data, in this case children’s growth charts, only when the child has recently developed well:

I always offer them to see the [growth] chart, sometimes they are very interested, and sometimes they are not. Depends, I guess, on whether they’ve done well or if their child is losing weight. Sometimes they feel a little bit like, no, we don’t want to see it today, but when they’ve done really well they want to see the child, you know this is where we’re going.

C2

Providing patients who have an avoidance coping strategy with test results and medical information can both decrease and increase the level of distress they experience. The timing and mode of information provision should be adapted to the patient and designed carefully. While clinicians might be able to judge people’s ability to take in such information, a PCEHR design has to consider scenarios in which patients are overwhelmed by information upfront.

## Discussion

As discussed earlier, most of the literature on technology for patient empowerment implicitly assumes that patients are willing and waiting to take power and responsibility for their health. Our study has shown that this willingness depends heavily on the patient’s coping style and perceived competence, autonomy, and relatedness. These findings match with existing theory on coping styles, on self-determination (SDT), and with the work by Aujoulat et al [6] on patient empowerment. In this section, we propose ways of applying these theories to the design of future technology for patient empowerment that will meet patients’ needs better.

### Theory on Coping Styles

In our study, the way patient families coped with the chronic condition of their child strongly influenced their PCEHR use: The controller made extensive use of the PCEHR but the clinical team neither noticed nor benefitted from their engagement; according to their clinicians, collaborators made excellent use of the PCEHR and cooperated with clinicians in a very helpful way; the cooperators felt comfortable using the PCEHR but generally chose to use it less than the collaborators; and the avoiders barely used the PCEHR at all. Based on this analysis, we conclude that patient families with different coping styles have different user needs that need to be considered when designing PCEHRs, and these differences extend far beyond simple information needs.

By employing theory on coping styles for the design of patient empowerment technology, we can both help to ensure that a specific coping style is supported by the technology and help patients to cope more effectively. While the technology should respect the needs of patients with different coping styles, there are potential benefits in helping patient families to develop an approach-oriented coping style, as this style has been found to be more effective in limiting psychological distress [46].

SDT [26] provides a promising approach to the development of intrinsic motivation which, in turn, is a driver for approach-oriented coping. In the following section, we identify possibilities to incorporate the principles of SDT in PCEHR design.

### Theory on Self-Determination and Intrinsic Motivation

We are not the first to use SDT [26] to design interventions within health promotion and health care contexts or understand their effectiveness. Ng et al [47] conducted a meta-review of 184 studies that applied SDT to the health care context. Their review confirmed the expected relations among the SDT variables and positive relations of psychological need satisfaction and autonomous motivation to beneficial health outcomes. Furthermore, they found a positive association between a supportive health care climate, better mental health, self-regulated behavior, quality of life, and satisfaction of all 3 psychological needs (competence, autonomy, and relatedness). Consistently, controlling health care climates results in people feeling undermined, with low motivation and sense of well-being.

#### Competence

By conducting a path analysis with the reviewed studies, Ng et al [47] found that competence explained the largest proportion of the variance in health outcomes. According to them, this result highlights that feeling competent is imperative for making the right behavior changes.

Looking at health management technology and PCEHRs in particular, patients are faced with 2 types of competence to acquire, namely, technological competence and disease-related competence.

Not feeling competent and comfortable using the technology can be a barrier even for patients who are in general comfortable using technology. Contributing to or editing a PCEHR may seem frightening, as patients may believe that any mistake can cause severe consequences for their health and treatment. In our study, the controlling patient family, P11, for example, was afraid of entering data incorrectly. The PCEHR stored the author of every data item, so that clinicians can check that the information is reliable. However, this feature was not obvious for this patient family. This highlights a need for the user interface to *make data security mechanisms very clear to users*, so that they feel comfortable to explore and to learn about the technology. Furthermore, *adequate introduction* and training are essential, and *ease of use* is of paramount importance.

PCEHRs can foster disease-related competence by providing general and personal medical information easily, securely, and efficiently. Patient families in our sample did not express a need for more information. However, 5 patient families mentioned that they had needed more information immediately after diagnosis. When technology is used to inform and educate patients, the information provided has to be relevant for the patients and fit their current level of knowledge and competence. Some researchers have investigated the variance of patients’ information needs. Attfield et al [18] investigated how patients’ information needs vary over time, specifically before and after consultations, and Al-Busaidi et al [12] presented an approach to providing patients with personalized and comprehensible information about their condition. They designed a patient portal that linked data from a patient’s medical record with relevant information on the Web and presented that information in patient-adequate language. These investigations provide first insights into ways to tailor to different information needs. Future work could investigate how such systems could also take patients’ previous knowledge and perceived competence into account.

#### Autonomy

The second basic need that SDT identifies is autonomy. Providing patients with access to their health record was found to increase their sense of autonomy [48]. One explanation for this effect is that giving patients access to their data allows them to reflect on it, to draw their own conclusions, and to make their own decisions [49].

On the contrary, nudging patients to use a PCEHR could decrease their perceived autonomy, causing anxiety and distress [47]. Indeed, Ng et al [47] found that positive results obtained with pressure to use a PCEHR are often short term. Concluding, the use of a PCEHR and all its features has to be voluntarily (as it was in this study). However, the effects of autonomy on nonadherence (eg, when a patient chooses not to adhere to a recommendation) would benefit from future research [47].

#### Relatedness

The third basic need of SDT is relatedness. Ng et al [47] confirmed that all 3 basic psychological needs predicted indicators of patient welfare. Relatedness correlated with an autonomy-supportive health care climate. In our study, the controlling patient family (P11) had had negative experiences with their health care providers. One way to prevent this family from experiencing distress or taking too much control might be to validate their negative experiences and to work with them to reframe it in a way that promotes relatedness.

A PCEHR could contribute to a positive health care climate if it allows patients to communicate their needs, values, and personal illness narratives to clinicians involved in their care. Furthermore, a PCEHR can connect all people involved in the patient’s care as well as patients in a similar situation who are looking for exchange and support. The potential and benefits of digital support networks have been demonstrated by others [50]. In our study, participants did not explicitly mention the need to connect to people other than their health care providers through the PCEHR, but the potential benefits of facilitating social support through PCEHRs should be investigated in future research.

### Theory on Taking and Relinquishing Control

Aujoulat et al [6] argue that the process of relinquishing control is as necessary as gaining control for patient empowerment. A strong sense of mastery and a feeling of control can sometimes even indicate that a patient is avoiding awareness of the impact illness has on his/her life [6]. Technologies for patient empowerment are usually designed to foster the feeling of mastery and control. However, in line with the findings of Aujoulat et al [6], our study shows that these technologies have to support the process of relinquishing control as well. We have identified 2 ways in which PCEHRs might help patients to relinquish control.

First, as noted by Aujoulat et al [6], understanding and accepting the cause of the illness can help with relinquishing control. Therefore, one approach is to provide patients with enough information to comprehend the rationale behind diagnosis and treatment, and to understand that they are not to blame for the diagnosis and that their control over the disease is limited.

Second, technology for patient empowerment might enable patients to articulate their needs for support: patients could communicate their needs and values in the PCEHR, share their illness narrative with their health care providers, request support, and explicitly choose not to take control and responsibility for a part of the treatment. In some cases, this may mean that patients elect not to receive information (eg, test results) before their next clinic appointment: for some patients, information can increase anxiety, when the patient is not prepared to take responsibility for interpreting that information independently of their clinical team. If self-awareness and choice are central themes in patient empowerment, the conscious decision not to take control of some aspects of an illness can itself be empowering.

### Study Limitations

For reasons outlined earlier, the number of participants in some groups (particularly the outlier) was small; however, in terms of theoretical constructs, the study maps closely onto constructs that have previously been identified by others (while highlighting their significance in a new study context), giving confidence that the findings are likely to generalize beyond this particular study population.

The study was conducted across 2 departments of one children’s hospital. Apart from participant 10, who was a patient under the care of the hospital, all participants were families; in most cases, the mother was the principal participant. Because the findings resonate with those of earlier studies on patient empowerment [6] and on patients’ coping styles, it is likely that these findings would generalize to patients as well as families, but this should be verified through a complementary study in an adult hospital, with individuals who are managing their own health. Another area for future research is the transition period (ie, when parents relinquish control to the patient), and how the PCEHR can support that transition.

The controlling patient family (P11) had had negative experiences with health care providers. Future research is needed that investigates whether patients with a different coping style, for example, collaborators or cooperators, would adopt similar behavior if they experienced a significant negative health event like a clinical error.

Participants were all families in which a child was suffering from a chronic and complex condition, under the care of a specialist children’s hospital. Many of the children involved were under the care of multiple clinicians across several sites (eg, General Practitioner/Primary Care practitioner, local hospital, specialist hospital), so their needs for care, and for coordination of care, are at an extreme of complexity. Consequently, participants might be expected to have strong motivations to engage with a PCEHR that helps with managing that complexity; also, most of them were highly experienced at coping with their child’s condition. The distribution of coping strategies and ways of engaging with a PCEHR might be different in a user population with less complex conditions, or conditions of shorter duration. Nevertheless, we have no reason to believe that the relationship between coping style and engagement with the PCEHR would differ substantively from that found in this study.

Patient families’ coping, and consequently their user needs, are likely to vary over the course of a disease. A patient who was recently diagnosed might, for example, have a greater need for explanatory information than a patient who has lived with a disease for many years. As our study was conducted in 3 months, we were not able to gather data about changing user needs; this is an area for future work.

While previous work suggested that computer, reading, or health literacy influence the adoption or nonadoption of a PHR [44], in our data the patient’s adjustment toward the disease correlated with motivation to use the PCEHR. Our data do not support the level of analysis necessary to discard or confirm the influence of participants’ literacy; again, this is an area for future work.

### Conclusion

In summary, in this study, we found that not all patient families are willing to take more control and responsibility in their health management, or motivated to use technology that is meant to empower them.

An important source of differences in patient families’ needs and wants was found to be their coping styles. Approach-oriented people were found to use the PCEHR heavily to track symptoms, medication, and food intake and to investigate test results. By contrast, avoidance-oriented people used the PCEHR only when necessary to coordinate care or to communicate with the clinical team, or did not use it at all.

Importantly, extensive use of a PCEHR did not necessarily indicate that a patient family felt empowered. As noted by Aujoulat et al [6], true patient empowerment necessitates both taking and relinquishing control. However, motivation to take control is only empowering if it is intrinsic: that is, if basic needs for competence, autonomy, and relatedness are fulfilled.

The focus of this study has been on PCEHRs, which can increase motivation to take responsibility in health management, potentially allowing people to better understand causal relations between treatments, other actions, and outcomes, and to identify opportunities for improvement. Timely access to health information also gives people, who are experts in managing their own (or their child’s) care, the opportunity to see if they can find something the doctors are missing.

Looking to the future, patient empowerment interventions should be systematically designed to meet people’s needs in managing care. In the study reported here, coping style was identified as an important attribute that needs to be taken into account in designing and deploying interventions. It is not sufficient to “activate” patients as if all patients respond in the same way to being given access to information and responsibility for managing care. The challenge is to tailor future systems to meet patients’ (and families’) needs, including their needs for autonomy, competence, and relatedness. We have outlined possible approaches to addressing these needs, while also highlighting areas for future study.

## Abbreviations

PCEHR: patient-controlled electronic health record
PHR: personal health record
PN: parenteral nutrition
SDT: Self-Determination Theory
